# Neurally Released GABA Acts via GABA_C_ Receptors to Modulate Ca^2+^ Transients Evoked by Trains of Synaptic Inputs, but Not Responses Evoked by Single Stimuli, in Myenteric Neurons of Mouse Ileum

**DOI:** 10.3389/fphys.2018.00097

**Published:** 2018-02-13

**Authors:** Katerina Koussoulas, Mathusi Swaminathan, Candice Fung, Joel C. Bornstein, Jaime P. P. Foong

**Affiliations:** Department of Physiology, University of Melbourne, Parkville, VIC, Australia

**Keywords:** GABA, GABA receptors, enteric nervous system, synaptic transmission, myenteric plexus

## Abstract

γ-Aminobutyric Acid (GABA) and its receptors, GABA_A,B,C_, are expressed in several locations along the gastrointestinal tract. Nevertheless, a role for GABA in enteric synaptic transmission remains elusive. In this study, we characterized the expression and function of GABA in the myenteric plexus of the mouse ileum. About 8% of all myenteric neurons were found to be GABA-immunoreactive (GABA+) including some Calretinin+ and some neuronal nitric oxide synthase (nNOS+) neurons. We used *Wnt1-Cre;R26R-GCaMP3* mice, which express a genetically encoded fluorescent calcium indicator in all enteric neurons and glia. Exogenous GABA increased the intracellular calcium concentration, [Ca^2+^]_i_ of some myenteric neurons including many that did not express GABA or nNOS (the majority), some GABA+, Calretinin+ or Neurofilament-M (NFM)+ but rarely nNOS+ neurons. GABA+ terminals contacted a significantly larger proportion of the cell body surface area of Calretinin+ neurons than of nNOS+ neurons. Numbers of neurons with GABA-induced [Ca^2+^]_i_ transients were reduced by GABA_A,B,C_ and nicotinic receptor blockade. Electrical stimulation of interganglionic fiber tracts was used to examine possible effects of endogenous GABA release. [Ca^2+^]_i_ transients evoked by single pulses were unaffected by specific antagonists for each of the 3 GABA receptor subtypes. [Ca^2+^]_i_ transients evoked by 20 pulse trains were significantly amplified by GABA_C_ receptor blockade. These data suggest that GABA_A_ and GABA_B_ receptors are not involved in synaptic transmission, but suggest a novel role for GABA_C_ receptors in modulating slow synaptic transmission, as indicated by changes in [Ca^2+^]_i_ transients, within the ENS.

## Introduction

γ-Aminobutyric Acid (GABA), a prominent neurotransmitter in the central nervous system (CNS) is expressed by neurons of the enteric nervous system (ENS), enteroendocrine cells in the mucosa and nerve terminals in the smooth muscle layers of the gastrointestinal tract (Jessen et al., 1986; Krantis et al., 1994; Sang and Young, 1996; Krantis, 2000). GABA has been proposed to be a neurotransmitter in the ENS for decades, as early studies on the guinea-pig intestine showed that GABA can excite enteric neurons (Krantis et al., 1980; Cherubini and North, 1984a,b), and that electrical field stimulation of cultured enteric neurons releases [^3^H]GABA (Jessen et al., 1983). Nonetheless, its role in synaptic transmission has remained elusive.

GABA is expressed in the ENS of several species; but significant interspecies differences in the distribution of GABA neurons and fibers have been reported (Furness et al., 1989; Messenger and Furness, 1990; Sang and Young, 1996; Timmermans and Scheuermann, 1998; Krantis, 2000). Cell bodies of GABA-containing neurons are predominantly found in the myenteric plexus of the ENS and have terminals in the myenteric plexus and the longitudinal and circular muscle, and so they are thought to be interneurons and motor neurons (Hills et al., 1987; Furness et al., 1989; Sang and Young, 1996). In the large intestine of rats and mice, GABA is expressed in the cell bodies of 5–14% of all myenteric neurons (Sang and Young, 1996; Krantis, 2000), while GABA cell bodies comprise about 5% of all myenteric neurons in the guinea-pig and mouse small intestine (Williamson et al., 1996; Li et al., 2011). In the mouse small intestine, expression of GABA is reported to be sparse and hence excluded from co-localisation studies (Sang and Young, 1996, 1998). GABA may have a role in synaptic transmission in this region as there are many GABA-immunoreactive myenteric terminals in the mouse small intestine (Jessen et al., 1986; Hills et al., 1987; Sang and Young, 1996), but these have not been examined in depth.

All three major subtypes of GABA receptors have been identified within the ENS. GABA_A_, GABA_B_, and GABA_C_ receptors are all expressed in the rat small and large intestine (Krantis et al., 1995; Nakajima et al., 1996; Poulter et al., 1999; Fletcher et al., 2001). In mouse, GABA_A_ receptor subunits and GABA_B_ receptors are expressed in the large intestine (Casanova et al., 2009; Seifi et al., 2014); the presence of the 3 receptor subtypes in the small intestine has been deduced from functional contractility studies (Sanger et al., 2002; Zizzo et al., 2007) although GABA_C_ receptor expression and function has been particularly elusive in comparison to its counterparts (Auteri et al., 2015).

Unlike its predominant inhibitory role in the CNS, GABA can have both an excitatory and inhibitory influence on enteric neurons depending on the receptor it activates. GABA can act through GABA_A_ and GABA_C_ receptor subtypes, which are both pentameric chloride channels, to excite enteric neurons (Auteri et al., 2015). This due to the high intracellular Cl^−^ concentration in enteric neurons, which is maintained by a Na^+^-K^+^-2Cl^−^ symporter (Liu et al., 2013). In contrast, activation of GABA_B_ receptors causes neuronal inhibition, as they are metabotropic G protein-coupled receptors that inhibit presynaptic voltage-activated Ca^2+^ channels and activate postsynaptic inwardly rectifying K^+^ channels (Auteri et al., 2015). The net functional output of GABA is difficult to predict as it depends on the receptor subtypes involved and their species- and region-specific localization (Auteri et al., 2015). Even within the same gut region, varying concentrations of GABA can alter the receptor subtype that is recruited leading to changes in the behavioral output (Auteri et al., 2014). While several studies have reported modulatory roles for GABA and its receptors on contractile activity in the gut (Tonini et al., 1989a,b; Sanger et al., 2002; Zizzo et al., 2007; Auteri et al., 2014), a role for GABA in synaptic transmission has not been identified.

In this study, we employed Ca^2+^-imaging from *Wnt1-Cre;R26R-GCaMP3* mice which express a genetically encoded fluorescent calcium indicator in all enteric neurons and glia as a high throughput assay to elucidate the elusive role of GABA in synaptic transmission in the murine small intestine. We identified myenteric neuronal subtypes that express functional GABA receptors via the use of specific GABA receptor antagonists and *post-hoc* immunohistochemistry. Our data suggest that GABA_A_ and GABA_B_ receptors do not have a synaptic function within the ENS, but demonstrate a novel role for GABA_C_ receptor in modulating slow synaptic transmission within the ENS.

## Materials and methods

### Experimental animals and tissue preparation

Mice of either sex from a C57Bl/6 background aged 8–12 weeks, including *Wnt1-Cre;R26R-GCaMP3* mice, in which neural crest-derived cells express the genetically encoded calcium indicator, GCaMP3 (Zariwala et al., 2012; Boesmans et al., 2013) were used. *Wnt1-Cre;R26R-GCaMP3* mice were the progeny of *Wnt1-Cre* mice (Danielian et al., 1998) and *R26R-GCaMP3* mice (The Jackson Laboratory). Mice were killed by cervical dislocation; a procedure approved by the University of Melbourne Animal Experimentation Ethics Committee. A segment of distal ileum, 2 cm proximal to the ileo-caecal junction, was removed from each mouse and immediately placed in physiological saline (composition in mM: NaCl 118, NaHCO_3_ 25, D-glucose 11, KCl 4.8, CaCl_2_ 2.5, MgSO_4_ 1.2, NaH_2_PO_4_ 1.0) bubbled with carbogen gas (95% O_2_, 5% CO_2_). The ileal segments were then cut along the mesenteric border, stretched and pinned flat mucosal side up in a Petri dish lined with a silicone elastomer (Sylgard 184; Dow Corning, North Ryde, NSW, Australia).

### Expression of GABA and other neuronal subtype markers

Pinned and stretched segments of ileum of mice from a C57/Bl6 background were fixed overnight in 4% formaldehyde in 0.1 M phosphate buffer, pH 7.2, at 4°C, and then the tissue was given three washes with phosphate-buffered saline (PBS). Longitudinal muscle-myenteric plexus (LMMP) whole-mount preparations were obtained by firstly removing the mucosa-submucosa layer, then stripping away overlying circular muscle via microdissection. These preparations were incubated for 30 min with 1% triton X-100 (ProSciTech, Thuringowa, QLD, Australia) at room temperature. The tissue was then given three washes with PBS, followed by overnight incubation with a combination of primary antibodies (Table 1) at 4°C. After three washes with PBS, the tissue was incubated with a combination of relevant secondary antibodies (Table 1) for about 2.5 h at room temperature. The tissue was given another three washes with PBS, and then mounted onto a slide with Dako fluorescent mounting medium (Carpinteria, Califonia, USA).

**Table 1:** Primary and secondary antisera used.

	**Raised in**	**Dilution factor**	**Source**
**PRIMARY ANTISERA**			
GABA	Rabbit	1:1000	Sigma Aldrich
nNOS	Sheep	1:1000	Gift from Dr P. Emson
Hu	Human	1:5000	Gift from Dr V. Lennon
Calretinin	Goat	1:1000	SWANT
NFM	Rabbit	1:500	Merck Millipore
**SECONDAY ANTISERA**			
Anti-rabbit AF 647	Donkey	1:400	Molecular Probes
Anti-sheep AF 594	Donkey	1:100	Molecular Probes
Anti-sheep AF 488	Donkey	1:400	Molecular Probes
Anti-human 594	Donkey	1:750	Jackson Immuno Labs

Preparations of myenteric plexus were viewed using a Zeiss Axio Imager M2 microscope and images were acquired with a Axiocam 506 mono camera using Zen 2.3 (blue edition) software (all from Zeiss, Australia). Images were taken using 20x or 40x objectives. The proportion of Calretinin and/or nNOS and/or GABA expressing neurons was obtained by examining co-expression with the pan-neuronal marker, Hu. GABA staining within neuronal cell bodies in this study was found to be highly variable, but unambiguous. Myenteric ganglia were imaged and a neuron was considered to be immunoreactive for GABA if the staining of its cytoplasm was higher than the background such that an unstained nucleus could be seen. At least 200 Hu+ cell bodies were counted in each preparation. The mean proportion of each neuronal subtype was determined by calculating averages from 3 animals. The data are expressed as mean ± SEM and n = the number of cells examined. Statistical analyses were performed using unpaired *t*-tests with *P* < 0.05 considered statistically significant. Comparisons were performed using using GraphPad Prism 5.0 (GraphPad Softwares, San Diego California).

To quantify GABA-immunoreactive terminals apposing Calretinin- and nNOS- immunoreactive neurons, high-resolution z-stacks of preparations co-stained with GABA and Calretinin or nNOS were imaged using a Zeiss LSM800 confocal microscope. The images were sampled at a resolution of 1,024 × 1,024 pixels using the 63x/1.40 Oil DIC M27 objective, with a 1.8x software zoom and z steps of 0.43 μm. Fourteen to seventeen Calretinin- or nNOS-immunoreactive neurons were examined from each preparation. A total of 3 preparations, each from a different animal, was examined. 3D rendered surfaces of the neuronal cell-bodies and GABA terminals were generated using the 3D image analysis software Imaris x64 (Bitplane, version 9.0.0). The percentage of total cell surface area covered by GABA-ergic terminals was determined for each Calretinin- or nNOS-immunoreactive neuron. Data are presented as the mean % surface area ± SEM, where n = number of neurons examined. Statistical analyses were performed using unpaired *t*-tests with *P* < 0.05 considered statistically significant. Comparisons were performed using using GraphPad Prism 5.0 (GraphPad Softwares, San Diego California).

### Calcium imaging

#### Tissue preparation

Segments of ileum were removed from *Wnt1-Cre;R26R-GCaMP3* mice, cut along the mesenteric border and pinned flat, mucosa side up in a dish lined with silicone elastomer (Sylgard 184; Dow Corning). The mucosa and submucous plexus were removed from the underlying smooth muscle and myenteric plexus layers, then strips of circular muscle were carefully peeled off and the resultant LMMP preparations were stretched over a small inox ring and immobilized by a matched rubber O-ring (Vanden Berghe et al., 2002). A maximum of 5 rings was prepared from each segment of ileum. The rings were transferred to an organ bath for imaging. The bath was constantly superfused (1 ml/min) with 95% O_2_, 5% CO_2_ physiological saline at room temperature throughout the experiment via a gravity-fed inflow system that included a manual valve to switch between saline and drug-containing saline solutions.

#### Imaging

The ring preparations were imaged using a 20x (NA 0.5) water dipping objective on an upright Zeiss Axioskope microscope with a Zeiss AxioCam MRm camera, and images (278 × 278) were acquired at 1 Hz.

Neurons within the myenteric ganglia were stimulated either chemically or electrically. Chemical stimulation involved the pressure ejection (spritz, 2 s duration; 9 psi) of GABA (1 mM) using a micropipette (tip diameter ~20 μm) situated right at the edge of the imaged ganglion. During time control experiments GABA was applied to each ganglion via 2 repeated local applications, each separated by a 10 min time period. In initial studies, the amplitudes of GABA-evoked [Ca^2+^]_i_ responses were significantly reduced at the second time point suggesting desensitization of receptors, in accordance with previous reports (Cherubini and North, 1984a,b). To prevent potential desensitization of GABA receptors, the spritz pipette was moved away from the ganglion in between applications. To electrically stimulate the neurons, a focal stimulating electrode (tungsten wire; 50 μm diameter) was placed on an interganglionic fiber tract leading into the ganglion of choice where a single pulse and a train of 20 pulses (20 Hz) were elicited. For time control experiments, ganglia were stimulated first with a single pulse and then a train of 20 pulses 5 min later. This stimulation regime was repeated 2 times 10 min apart.

To test the effects of antagonists, drug-containing saline was superfused for 10 min into the organ bath after either the first GABA spritz or electrical stimulation regime, so that the first spritz or electrical time point was taken as the control response. Each ringed preparation was only exposed to an antagonist, or a combination of antagonists, once.

Following live-imaging experiments, selected preparations tissues were fixed overnight with 4% formaldehyde at 4°C and immunostained using primary antisera to the neuron subtype markers, GABA, nNOS, neurofilament-M (NFM) and/or calretinin together with secondary appropriate antisera (Table 1), to identify the neurochemistry of the responding neurons.

#### Data analysis and statistics

Analyses were performed using custom-written directives in IGOR Pro (WaveMetrics, Lake Oswego, Oregon, USA). Regions of interest were drawn over a selected area of the cytoplasm for each neuron, excluding the nucleus because GCaMP3 is absent from the nuclei (Tian et al., 2009; Yamada and Mikoshiba, 2012). The intensity of the intracellular calcium ([Ca^2+^]_i_) transient signal for each response was calculated and expressed as the maximum increase in [Ca^2+^]_i_ from the baseline signal (ΔFi/F0). [Ca^2+^]_i_ transients were only considered if the intensity of the transient signal was more than 5 times the intrinsic noise. For both time control and antagonist experiments the ΔFi/F0 of the second GABA spritz, or second electrical stimulation response, was normalized and expressed as a fraction of the first (%ΔFi/F0). A minimum of 3 animals were examined for each experimental set; unless stated otherwise.

Ganglia of interest that were examined with Ca^2+^-imaging were processed for *post-hoc* immunohistochemistry and relocated using 20x or 40x objectives of a Zeiss Axio Imager M2 microscope. Images were acquired with a Axiocam 506 mono camera using Zen 2.3 (blue edition) software (all from Zeiss, Australia). Responding GCaMP3+ neurons were identified immunohistochemically by matching the micrographs with the Ca^2+^-imaging videos.

Data are presented as the mean % ΔF_i_/F_0_ of the control ± SEM where n = number of neurons examined or as mean ± SEM and n = the number of cells examined. Statistical analyses were performed using one-way analysis of variance (ANOVA) followed by Dunnett's *post-hoc* test with *P* < 0.05 considered statistically significant. Comparisons were performed using GraphPad Prism 5.0 (GraphPad Softwares, San Diego California).

#### Drugs used

Drugs used included GABA, hexamethonium bromide (both from Sigma Aldrich, Castle Hill, New South Wales, Australia), Bicuculline, [S-(R^*^,R^*^)]-[3-[[1-(3,4-Dichlorophenyl)ethyl]amino]-2-hydroxypropyl](cyclohexylmethyl) phosphinic acid (CGP 54626) and (1,2,5,6-Tetrahydropyridin-4-yl)methylphosphinic acid (TPMPA) (all from Tocris Bioscience, Avonmouth Bristol UK). All drugs were diluted in distilled water to make stock solutions and then again in physiological saline to their working concentration on the day of experimentation.

## Results

### Expression of GABA in the distal ileal myenteric plexus

**Figure 1:**
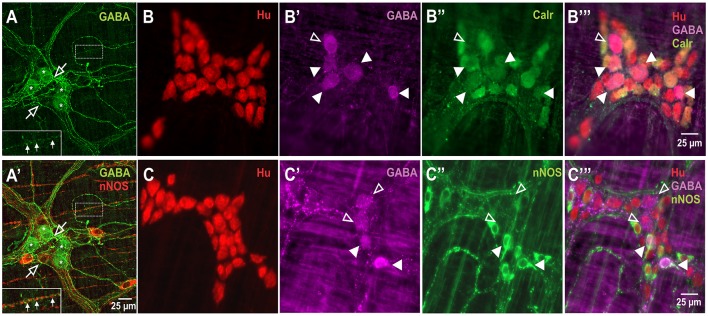
Expression of GABA in relation to Hu, Calretinin and nNOS stains in the myenteric plexus of the mouse ileum. Confocal micrographs of a myenteric ganglion showing GABA immunoreactivity in the somata (asterisks) of some non-nitrergic neurons, terminals and varicosities (open arrows) within the ganglion **(A,A')**. GABA and nNOS also stained different varicosities on muscle strips (**A,A'** inset, filled arrows). GABA **(B',B”',C',C”')**, Calretinin **(B”,B”')** and nNOS **(C”,C”')** immunoreactivity were found in some Hu+ neurons. Overall, GABA colocalised with some Calretinin+ and nNOS+ neurons. Example confocal micrographs showing GABA colocalisation with some Calretinin+ neurons **(B)**, and nNOS+ neurons **(C)** (colocalisation—filled arrowheads, no colocalisation—open arrowheads).

GABA+ neurons are relatively uncommon in the myenteric plexus of the mouse ileum and very little is known about their functional classification in this gut region (Sang and Young, 1996; Li et al., 2011). nNOS+ neurons have been described mainly as inhibitory motor neurons, while Calretinin+ neurons are intrinsic sensory neurons, interneurons and excitatory neurons to the muscle layers (Sang and Young, 1996; Li et al., 2011). In this study, GABA immunoreactivity was expressed in the somata of 8.2 ± 0.7% of Hu+ cells (*n* = 3 animals, Figure 1). GABA+ terminals and varicosities were also present within the myenteric ganglia and muscle layers (Figure 1A). nNOS immunoreactivity was observed in 24.3 ± 2%, while Calretinin immunoreactivity was found in 33 ± 1.4% of Hu+ neurons (both *n* = 3 animals, Figure 1). This is similar to previous reports in the mouse ileum (Sang and Young, 1996; Qu et al., 2008). Furthermore, of the total number of Hu-immunoreactive neurons examined, we found that GABA colocalised with some of the nNOS+ (2.0 ± 0.1%) and Calretinin+ (3.7 ± 0.6%) neurons.

### Exogenous application of GABA evoked [Ca^2+^]_i_ transients directly in half of the responding neurons, the vast majority of responding neurons do not express GABA or nNOS

**Figure 2:**
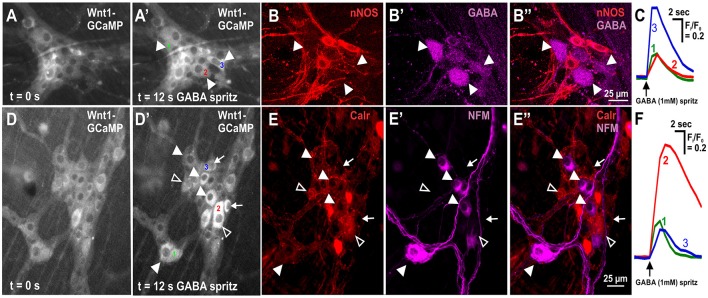
Neurochemistry of neurons in the mouse ileum that displayed GABA evoked [Ca^2+^]_i_ transients. **(A)** Representative fluorescence micrographs of the GABA (1 mM) evoked [Ca^2+^]_i_ response in myenteric neurons [GCaMP3 signal at rest (**A**, *t* = 0); and during GABA stimulation (**A'**, *t* = 12 s)]. **(A')** In this ganglion, 3 neurons responded to GABA with a sharp increase in [Ca^2+^]_i_ (responsive neurons marked 1–3). **(B)** Confocal micrographs of the myenteric ganglion showing the neurons that responded to GABA **(A')** were immunoreactive for GABA (filled arrowheads) and not nNOS. **(C)** Example traces from the 3 neurons (marked in **A'**) that responded to GABA. **(D)** Representative fluorescence micrographs of the GABA (1 mM) evoked [Ca^2+^]_i_ response in myenteric neurons [GCaMP3 signal at rest (**D**, *t* = 0); and during GABA stimulation (**D'**, *t* = 12 s)]. **(D')** In this ganglion, many neurons responded to GABA with a sharp increase in [Ca^2+^]_i_ (some marked with arrowheads and arrows, selected responsive neurons marked 1–3). **(E)** Confocal micrographs of the myenteric ganglion showing the neurons that responded to GABA **(D')** were immunoreactive for NFM (filled arrowheads), Calretinin (open arrowheads) or neither NFM or Calretinin (arrows). **(F)** Example traces from the selected 3 neurons (marked in **D'**) that responded to GABA.

GABA (1 mM) was spritzed onto 10 myenteric ganglia (from 6 animals) and induced a sharp increase in [Ca^2+^]_i_ (ΔF_i_/F_0_ = 0.23 ± 0.03, *n* = 63) in 21.8 ± 2.6% of GCaMP3+ cells. GCaMP3+ cells include neurons and glia, but the responding cells were probably neurons as they had cell body sizes (~20 μm diameter) similar to those of neurons (Gabella and Trigg, 1984) and typically responded instantaneously to stimuli with a sharp increase in [Ca^2+^]_i_; glia are smaller and tend to respond with a delayed and gradual increase in [Ca^2+^]_i_ (Boesmans et al., 2013). Some responding cells were identified via *post-hoc* immunohistochemistry, with the vast majority of all responding cells being neither GABA nor nNOS immunoreactive (GABA-/nNOS-, 72.0 ± 6.2%) or GABA+ (23.1 ± 7.1%) and rarely nNOS+ and almost never GABA+/nNOS+. The overall amplitude of calcium transients of GABA-/nNOS- responders (ΔF_i_/F_0_ = 0.24 ± 0.03, *n* = 47) did not differ from that of the other GABA+ and nNOS+ immunoreactive groups. However, nNOS+ responders exhibited significantly smaller response amplitudes than GABA+ responders, (GABA+: ΔF_i_/F_0_ = 0.21 ± 0.03, *n* = 11, nNOS+: ΔF_i_/F_0_ = 0.09 ± 0.02, *n* = 4, *P* < 0.05). Interestingly within each ganglion, most GABA+ neurons responded to GABA spritz (75.0 ± 12.0%), but nNOS+ neurons rarely did (3.4 ± 2.2%) (Figures 2A–C).

**Figure 3:**
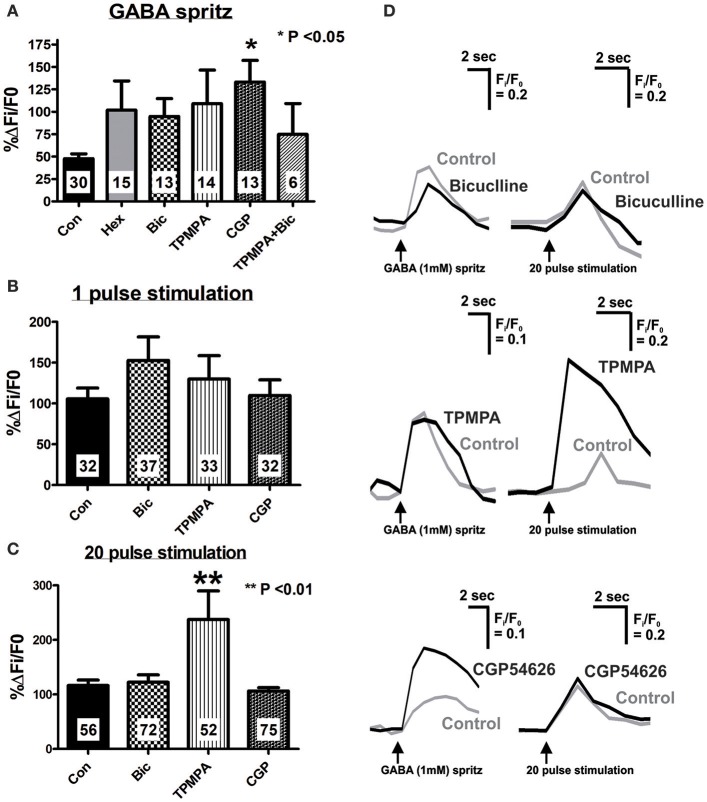
Effect of specific antagonists on GABA- and electrically-evoked [Ca^2+^]_i_ transients. Neurons were stimulated twice, first in the present of control saline, and second after 10 min application of antagonists (Hexamethonium, Hex 100 μM; Bicuculline, Bic 10 μM; TPMPA, 100 μM; CGP54626, CGP 1 μM or a combination of antagonists). Time controls were also performed (Con) where both stimuli were executed in control saline, 10 min apart. Histograms summarizing the pooled dataset from all neurons stimulated with GABA (1 mM) **(A)**, single pulse **(B)** or a train of 20 pulses **(C)**, excluding those that were abolished in the presence of antagonists (see Tables 2, 3). Changes in amplitude after application of various antagonists are presented as a percentage of the first response in control saline. **(A)** CGP54626 (GABA_B_ antagonist) significantly increased the [Ca^2+^]_i_ response evoked by GABA. **(B)** None of the specific GABA receptor antagonists had any effect on 1 pulse evoked [Ca^2+^]_i_ transients. **(C)** Only TPMPA had an effect on the 20 pulse evoked [Ca^2+^]_i_ transients, where it significantly increased the amplitude of the [Ca^2+^]_i_ response. **(D)** Example traces from a single neuron of GABA and 20 pulse-evoked changes in [Ca^2+^]_i_ in control conditions (gray traces) and in the presence of GABA receptor antagonists, (black traces).

Acetylcholine activating nicotinic receptors is the primary means of neurotransmission in the ENS (Nurgali et al., 2004; Gwynne and Bornstein, 2007; Foong et al., 2012, 2014). Addition of hexamethonium (nicotinic antagonist, 200 μM) to the superfusing solution abolished GABA-evoked [Ca^2+^]_i_ responses in 15/30 responding neurons (*P* = 0.008, Fisher's exact test; Table 2), and did not change the amplitude of GABA-evoked [Ca^2+^]_i_ responses of the hexamethonium resistant neurons (*n* = 15, *P* > 0.05; Figure 3A). Ten initially responsive neurons were identified by *post-hoc* immunohistochemistry: 8/10 were GABA-/nNOS-, 2 were GABA+. Of these neurons, the responses of the 3 neurons that were GABA-/nNOS- and both GABA+ neurons were abolished by hexamethonium.

**Table 2:** Number of neurons responding to GABA in control conditions and in the presence of antagonists.

**Time Point**	**1**	**2**
GABA spritz time controls	33	30
**Antagonists**	**Control**	**In the presence of drug/s**
Hexamethonium (nAChR, 200 μM)	30	15^∧^
Bicuculline (GABA_A_, 10 μM)	35	13^∧^
TPMPA (GABA_C_, 100 μM)	28	14^∧^
CGP54626 (GABA_B_, 1 μM)	20	13^*^
Bicuculline + TPMPA	22	6^∧^

* *P < 0.05*,

∧ *P < 0.001 Fishers exact test*.

### Calretinin neurons exhibit GABA evoked [Ca^2+^]_i_ transients, and have a larger proportion of their cell body surface area covered by GABA terminals than nNOS neurons

Calretinin and Neurofilament-M (NFM) are markers of excitatory and sensory neurons in the murine gut (Sang and Young, 1996; Qu et al., 2008). We found that the vast majority of GABA-responding neurons were GABA-/nNOS- and had large somata, which is suggestive of enteric sensory neurons (Qu et al., 2008). GABA (1 mM) was spritzed onto 6 myenteric ganglia (from 2 animals) and some GABA-responding neurons were found to be immunoreactive for NFM or Calretinin (Figures 2D–F). Twenty neurons responded to GABA with a sharp increase in [Ca^2+^]_i_; 16/20 were identified immunohistochemically with 9/16 being Calretinin-/NFM- and 7/16 being Calretinin+/NFM-. Of 36 neurons that responded to GABA in the presence of hexamethonium (200 μM), 35 were identified immunohistochemically: 15/35 were NFM-/Calretinin-, 9/35 were Calretinin-/NFM+, and 11/35 were Calretinin+/NFM-.

**Figure 4:**
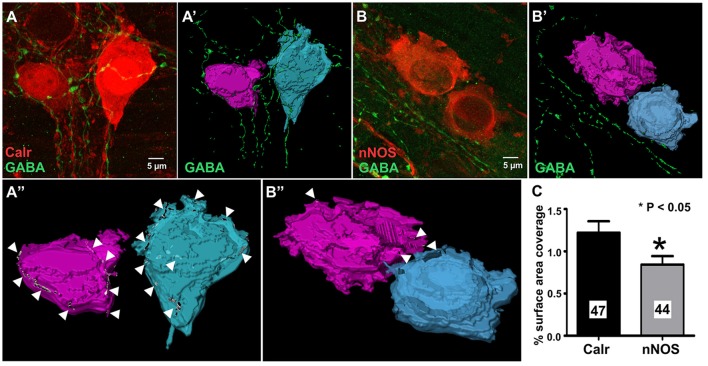
GABA terminals cover a larger proportion of the total cell surface area of 3D rendered calretinin- compared to nNOS- neurons. Example confocal micrographs of myenteric plexus stained for Calretinin and GABA **(A)**, and nNOS and GABA **(B)**. 3-D rendered surfaces of the calretinin- **(A')**, nNOS- **(B')** immunoreactive cell bodies, GABA-immunoreactive varicosities and terminals **(A',B')**. **(A”,B”)** Cell surface area receiving GABA terminals (arrow heads) detected by surface-surface contact analysis. **(C)** The mean percentage of total cell surface area covered by GABA terminals for Calretinin- or nNOS-immunoreactive neurons. Calretinin neurons have a significantly larger percentage surface cell body area that are covered by GABA terminals. All data were analyzed using the 3D image analysis software Imaris x64 (Bitplane, version 9.0.0).

Calretinin and nNOS immunoreactivity reveals the whole shape of the neuron (Figures 4A,B) and has been previously used to examine numbers of varicosities contacting their cell bodies using confocal microscopy (Neal and Bornstein, 2007). In this study, we used high resolution confocal microscopy and Imaris surface rendering software to quantify GABA-immunoreactive terminals and varicosities that apposed cell bodies of Calretinin- and nNOS- immunoreactive neurons. Unlike synaptic proteins such as alpha-synuclein, synaptophysin, synaptotagmin and synaptobrevin which only stain varicosities (Sharrad et al., 2013), in our preparations, GABA stained axons and varicosities (Figures 4A,B). This made accurate counts of individual GABA+ varicosities onto Calretinin and nNOS cell bodies difficult, so we analyzed the % surface area of the neuronal cell bodies that were contacted by GABA-ergic axons and varicosities instead. We found that the % surface area of Calretinin+ cell bodies that was contacted by GABA-ergic axons and varicosities was significantly greater than the contacted surface area of nNOS+ cell-bodies (% surface area contacted by GABA+ structures: Calretinin 1.2 ± 0.1, 47 neurons; nNOS 0.8 ± 0.1, 44 neurons; *P* = 0.03) (Figure 4).

### GABA evoked [Ca^2+^]_i_ transients are mediated through GABA A, B, and C receptors

The GABA_A_ receptor antagonist, bicuculline (10 μM, *P* = 0.0001, Fisher's exact test) and the GABA_C_ receptor antagonist, TPMPA (100 μM, *P* = 0.005, Fisher's exact test) significantly reduced the number of neurons that displayed GABA-evoked [Ca^2+^]_i_ responses (Table 2). The combined addition of bicuculline and TPMPA also abolished responses in a significant number of neurons (16/22 neurons, *P* = 0.0001, Fisher's exact test), but the proportion of neurons whose responses were abolished did not differ significantly from either antagonist alone (combination vs. bicuculline alone, *P* > 0.8; combination vs. TPMPA alone, *P* > 0.05, Fisher's exact test) (Table 2). The amplitudes of the responses in the remaining neurons were unchanged by GABA_A_ (*n* = 13, *P* > 0.05; Figures 3A,D) and GABA_C_ (*n* = 14, *P* >0.05; Figures 3A,D) blockade. Combined addition of bicuculline and TPMPA did not significantly alter the amplitudes of GABA-evoked [Ca^2+^]_i_ responses that were not abolished, (%ΔF_i_/F_0_ control: 48 ± 6 %, *n* = 30; %ΔFi/F0 bicuculline & TPMPA: 75 ± 38% *n* = 6, *P* > 0.1) (Figure 3A).

The GABA_B_ antagonist, CGP54626 (1 μM) abolished responses in a significant number of neurons that initially responded to GABA (*P* = 0.03, Fisher's exact test; Table 2). But amplitudes of [Ca^2+^]_i_ responses in the remaining neurons were significantly larger than control responses (*n* = 13, *P* < 0.05; Figures 3A,D).

### Involvement of endogenous GABA in synaptic transmission

Electrical stimuli consisting of a single pulse or a train of 20 pulses (20 Hz) were applied via a stimulating electrode to an internodal strand leading to the ganglion imaged, which are respectively expected to evoke fast-intermediate, and fast-intermediate-slow excitatory postsynaptic potentials in the ENS (Shuttleworth and Smith, 1999; Nurgali et al., 2004; Gwynne and Bornstein, 2007; Foong et al., 2012, 2015).

Bicuculline, TPMPA and CGP54626 did not affect the number of neurons that exhibited electrically-evoked [Ca^2+^]_i_ transients relative to time controls (all *P* > 0.1 Fisher's exact test; Table 3). The amplitude of single pulse-evoked [Ca^2+^]_i_ responses was unaffected by TPMPA (*n* = 33, *P* > 0.4; Figure 3B), but the amplitudes of train-evoked [Ca^2+^]_i_ transient responses were significantly potentiated by TPMPA (*n* = 52 *P* < 0.01; Figures 3C,D). Bicuculline and CGP54626 had no effect on the amplitudes of single or train-evoked responses (all *P* > 0.1; Figures 3B–D).

**Table 3:** Number of neurons responding to electrical stimulation in control conditions and in the presence of antagonists.

**Time Point**	**1**	**2**
1 pulse stimulation time controls	42	32
20 pulse train stimulation time controls	58	56
**Antagonists**	**Control**	**In the presence of drug/s**
Bicuculline (GABA_A_, 10 μM) 1 pulse stimulation	49	37
Bicuculline (GABA_A_, 10 μM) 20 pulse stimulation	79	72
TPMPA (GABA_C_, 100 μM) 1 pulse stimulation	41	33
TPMPA (GABA_C_, 100 μM) 20 pulse stimulation	60	52
CGP54626 (GABA_B_, 1 μM) 1 pulse stimulation	38	32
CGP54626 (GABA_B_, 1 μM) 20 pulse stimulation	79	75

*All P > 0.1 Fisher's exact test*.

## Discussion

The physiological roles of GABA and its receptors in the gastrointestinal tract have proved elusive as they can have excitatory or inhibitory effects on enteric neurons and their expression is highly specific to gastrointestinal regions and species (Auteri et al., 2015). In this study, we used a multifaceted approach to examine the role of GABA and its receptors in the myenteric plexus of the murine ileum. We characterized the expression of GABA in neurons and varicosities surrounding enteric neurons, and identified the neuronal subtypes that express GABA receptors using specific antagonists and Ca^2+^-imaging. We showed that GABA receptors are unlikely to mediate fast or intermediate EPSPs and revealed a novel role for GABA_C_ receptors activated by endogenously released GABA in modulating slow excitatory neurotransmission in the ENS.

### GABA colocalises with some calretinin and nNOS neurons in the myenteric plexus of the mouse ileum

In accordance with previous studies (Sang and Young, 1996; Li et al., 2011), we found that GABA neurons constitute 8% of all myenteric neurons in the murine ileum. Further, we found that GABA colocalised with a proportion of nNOS-immunoreactive neurons, which are often interneurons and inhibitory motor neurons, and some Calretinin-immunoreactive neurons which include intrinsic sensory, excitatory interneurons and motor neurons in the murine small intestine (Qu et al., 2008). Thus, GABA neurons may include subpopulations of such neurons. Indeed, we found GABA-immunoreactive varicosities in the circular muscle and within ganglia, where they closely apposed Calretinin-immunoreactive neurons and some nNOS-immunoreactive neurons. Thus, some GABA-containing neurons are probably interneurons contacting excitatory and inhibitory motor neurons as previously suggested for the rat colon (Krantis, 2000). This is consistent with findings from the murine colon, where widespread GABAergic innervation of neurons throughout the myenteric plexus has been identified by immunolabelling of the vesicular GABA transporter (vGAT) (Seifi et al., 2014).

### Myenteric neuronal subtypes that respond to GABA in the mouse ileum

Exogenous application of GABA evoked [Ca^2+^]_i_ transients in some myenteric neurons. The vast majority of these neurons were GABA-/nNOS-, including some Calretinin- and NF-M-immunoreactive neurons. nNOS-immunoreactive neurons rarely responded to GABA. Moreover, while GABA+ terminals and varicosities made close contacts to many Calretinin+ and nNOS+ neurons, the % surface area of Calretinin+ cell bodies contacted by GABA-ergic terminals was significantly greater than the contacted area of nNOS+ cell bodies. Although most GABA-immunoreactive neurons responded to GABA, these neurons made up only a small proportion of all responding neurons, reflecting their overall numbers. Nonetheless, we cannot exclude the possibility that GABA neurons make functional contacts onto other myenteric neurons including nNOS+ neurons that we did not detect.

Earlier intracellular studies of guinea-pig ileum showed that GABA excites intrinsic sensory myenteric neurons (which display distinctive AH-type electrophysiology) via GABA_A_ receptors. Conversely, all other types of neurons that display S-type electrophysiology, which includes interneurons and motor neurons, were insensitive to GABA (Cherubini and North, 1984a). Our study is congruent with this, as some neurons that expressed Calretinin and NF-M, both markers of intrinsic sensory neurons, responded to GABA (Qu et al., 2008). But Calretinin also labels excitatory interneurons and motor neurons, and some nNOS and GABA neurons responded, albeit in smaller numbers. Moreover, half the GABA-evoked responses were in secondary neurons of the circuitry as they were blocked by, hexamethonium indicating involvement of a cholinergic synapse. That neurons other than intrinsic sensory neurons respond to GABA, differs from the earlier findings in the guinea-pig ileum (Cherubini and North, 1984a), which may be due to differential GABA receptor expression, which has not been detailed across species.

### GABA_**A**_, GABA_**B**_, and GABA_**C**_ receptors are expressed in the myenteric circuitry

GABA-mediated chloride conductance via GABA_A_ and GABA_C_-receptors is known to produce depolarisations in enteric neurons, while GABA_B_ receptor activation causes neuronal inhibition (Liu et al., 2013; Auteri et al., 2015). We found that GABA_A_, GABA_B_, and GABA_C_ antagonists each prevent GABA-evoked calcium responses in many neurons. This suggests that some myenteric neurons, including nearly all GABA-immunoreactive neurons possess GABA_A_, GABA_B_, and GABA_C_ receptors; some may possess more than one subtype. The concentrations of GABA receptor antagonists used were taken from previous publications (Sanger et al., 2002; Zizzo et al., 2007; Auteri et al., 2014) that showed selectivity in functional studies. However, a point to consider is that GABA antagonists have non-specific effects. In particular, bicuculline can block nicotinic receptors (Zhang and Feltz, 1991) and can act on SK (small conductance) Ca^2+^-activated K^+^ channels (Debarbieux et al., 1998; Khawaled et al., 1999; Johansson et al., 2001). Each of these actions may have implications for neuronal excitability. However, such “off-target” effects cannot account for the failure of bicuculline to alter the responses to electrical stimulation of interganglionic connectives, as nicotinic blockade would depress such responses (Foong et al., 2015) and there is no evidence for involvement of SK channels in the regulation of enteric neuron excitability. Further, at higher concentrations (~EC_50_ 500 μM), TPMPA may weakly activate GABA_B_ receptors (Ragozzino et al., 1996), but as 5-fold lower concentrations were used in this study, an action on GABA_B_ receptors is unlikely.

A response to GABA that was not blocked by either GABA_A_ or GABA_C_ antagonists or both was identified, potentially mediated via GABA_B_ receptors. GABA_B_ receptor blockade abolishing responses in a significant number of neurons is a surprising finding, but application of a GABA_B_ receptor agonist induced [Ca^2+^]_i_ transients in cultured guinea-pig myenteric neurons (Reis et al., 2006). It is also possible that GABA_B_ receptors are located on inhibitory inputs to the responding neurons, such that when they are blocked, inhibition is restored thereby decreasing the number of GABA responders.

Responses that were not abolished by a GABA_B_ receptor antagonist were significantly larger than control responses, revealing a potential inhibitory action of GABA. Indeed GABA_B_ receptors in the mouse gut act at the presynapse to prevent ACh release from excitatory cholinergic neurons (Sanger et al., 2002; Hyland and Cryan, 2010), which may account for the increase in neuronal activity observed following GABA_B_ receptor blockade in this study.

In the mouse gut, GABA_A_ and GABA_B_ receptors have been identified in the colon (Casanova et al., 2009; Seifi et al., 2014; Auteri et al., 2015) and GABA_A_ receptors are expressed on neurochemically diverse cell types including nNOS, ChAT, 5-HT, and SOM neurons (Seifi et al., 2014). Expression of GABA_C_ receptors in the mouse was only deduced by contractility studies in the longitudinal muscle of the small intestine (Zizzo et al., 2007). We attempted to examine expression of GABA receptors on enteric neurons using immunohistochemistry, but failed to obtain satisfactory immunostaining with the available antisera. Thus, this component of the study was not pursued further.

### Involvement of endogenous GABA in synaptic transmission and a role for GABA_**C**_ receptors

Electrical stimulation to evoke endogenous neurotransmitter release was used in this study to ascertain whether any GABA receptors and therefore endogenous GABA contribute to synaptic transmission. Using a reporter mouse which expresses GCaMP3 in a Cre-dependent manner in the *Rosa26* locus (Tian et al., 2009; Zariwala et al., 2012) has revolutionized research into the examination of ENS excitability, allowing high-throughput analysis in previously challenging or impossible situations (Foong et al., 2015; Hao et al., 2017). With the omission of tissue loading steps, tissue viability and resolution are increased, background signals are reduced with the GCaMP3 indicator being specifically Wnt-1 driven to be selectively expressed in neural crest derivatives (enteric neurons and glia) within the heterogenous preparation (Boesmans et al., 2013). GCaMP3, although not the current fastest variant of genetically encoded calcium indicators in its family, can detect calcium transients with amplitudes linearly dependent on action potential number in the brain of intact mice (Tian et al., 2009). The stimulus regime and sampling frequency we used will detect [Ca^2+^]_i_ transients due to neuronal activity. While the relatively slow inactivation kinetics of GCaMP3 will not resolve fast events like action potentials and subthreshold fast EPSPs (Tian et al., 2009; Michel et al., 2011), we can detect their longer term consequences as neuronal activity. The recorded single pulse-evoked [Ca^2+^]_i_ transients in our study include responses to fast-intermediate synaptic events that are mediated by several neurotransmitters, but particularly include acetylcholine activating nicotinic receptors, the typical fast neurotransmitter in the ENS (Foong et al., 2015). While [Ca^2+^]_i_ transients evoked by a 20 Hz train of pulses include responses to neurotransmitters mediating fast, intermediate and slower synaptic events.

In our study, single pulse-evoked [Ca^2+^]_i_ responses were unaffected by any of the GABA receptor antagonists. Responses evoked by trains of stimuli were unaffected by GABA_A_ or GABA_B_ blockade, but potentiated by the GABA_C_ antagonist, TPMPA, which has a strong selectivity (>100-fold) for GABA_C_ receptors compared with GABA_A_ or GABA_B_ (Ragozzino et al., 1996; Johnston, 2002). Hence despite neural expression of all 3 GABA receptors, our data do not support a role for GABA in fast neurotransmission, but indicate that release of endogenous GABA modulates slow synaptic transmission, where GABA acting at GABA_C_ receptors tonically inhibits the system. The exclusion of GABA_A_ and GABA_B_ receptors is surprising as there is strong evidence in the guinea-pig myenteric plexus that GABA excites neurons via GABA_A_ receptors (Cherubini and North, 1984a,b, 1985; Zhou and Galligan, 2000), and in cultured guinea-pig myenteric neurons, application of GABA and agonists for GABA_A_ and GABA_B_ receptors induced [Ca^2+^]_i_ transients, while GABA_C_ agonist only elicited small responses in fewer neurons (Reis et al., 2006). Moreover, several studies have implicated functional roles for GABA_A_ and GABA_B_ receptors (Sanger et al., 2002; Zizzo et al., 2007; Auteri et al., 2014; Seifi et al., 2014), but there is only one report of involvement of GABA_C_ receptors (Zizzo et al., 2007) in the control of smooth muscle contractility in the mouse gut. While interspecies and gut regional differences can be postulated, we cannot exclude a potential role of GABA_A_ and GABA_B_ receptors in synaptic transmission, as [Ca^2+^]_i_ is only one indication of neuronal excitability so their contribution may be revealed using other types of measurements, sampling frequencies or stimulus regimes.

While GABA_C_ receptors mediate excitation via a depolarising conductance, intrinsic differences in receptor kinetics may account for their inhibition of responses to trains of stimuli. GABA_A_ receptors mediate rapid transient currents, while GABA_C_ receptors produce more prolonged responses (Qian and Dowling, 1995; Lukasiewicz and Shields, 1998), where the deactivation rate or closing phase of GABA_C_ receptors is much slower (Amin and Weiss, 1994). In addition, unlike GABA_A_ receptors, GABA_C_ receptors display little desensitization (Matthews et al., 1994). GABA_C_ receptors, like those located on terminals of retinal bipolar cells produce a (tonic) standing current due to these properties (Hull et al., 2006; Palmer, 2006). Slow excitatory transmission in intrinsic sensory neurons displaying AH-type electrophysiology, is associated with a net decrease in membrane conductance due largely to inhibition of two potassium conductances; resting or “leak” K^+^ conductance (gK) and calcium-dependent K conductance (gK_Ca_) (Grafe et al., 1980). While this applies to AH neurons, the situation involving S neurons is more complex as the K^+^ channels have not been clearly identified and there is potential for contribution by M- current channels like the KCNQ (Kv7) channels (Brown and Passmore, 2009; Hirst et al., 2015). In addition, slow transmission may also be due to an increase in chloride conductance (gCl) in myenteric neurons (Bertrand and Galligan, 1994). Inactivation of resting gK and activation of gCl leads to depolarisation of the neuron relative to the resting potential to a membrane potential approximating the reversal potential of Cl^−^ which is estimated to be between −39 (Cherubini and North, 1984a) and −17 mV (Bertrand and Galligan, 1994) in guinea-pig myenteric neurons. Therefore, activation and prolonged opening of GABA_C_ receptors and thus chloride conductance, would lead to a new membrane potential approximating the reversal potential of Cl^−^. The initial depolarisation of the cell at this membrane potential is likely to activate delayed rectifier K^+^ channels which are active when the membrane is depolarised to values above −30 mV (Hirst et al., 1985), producing a repolarising inhibition. This stabilization of the membrane would prevent the activation of voltage gated Na^+^ channels and further depolarisation, therefore allowing GABA to shunt action potential firing by a “clamping” of the membrane potential. Thus, following repetitive stimulation of interganglionic connectives, prolonged opening of these channels by endogenous GABA may be responsible for this shunting inhibition which would be released with GABA_C_ receptor blockade, producing the potentiated response observed in this study.

## Conclusions

Our study extended understanding GABA within the murine myenteric plexus in the small intestine, showing that some GABA-immunoreactive neurons are probably interneurons as in other species. Broadly surveying the ENS via Ca^2+^-imaging has enabled us to observe the previously elusive endogenous effects of GABA, to show expression of all 3 GABA receptor subtypes, and for the first time reveal a role for modulating synaptic transmission in the myenteric plexus via GABA_C_ receptors. The specific actions of GABA_C_ receptors will require more detailed analysis of the membrane potential, which would be via intracellular recording. However, recording individual neurons via sharp electrodes from a sufficient sample size of neurons is technically difficult and outside the scope of study. Predictions of neuronal behavior via realistic computational modeling experiments can also be employed in the future to examine the net effect of the activation of various receptors at neuronal synapses.

## Author contributors

KK, MS, JB, and JF conceived and designed the experiments. KK, CF, MS, and JF performed the experiments. KK, MS, and JF analyzed the data. JB and JF contributed reagents, materials, analysis tools. KK, JB, and JF wrote the manuscript. All authors contributed to editing and revising the manuscript. All authors read and approved the final manuscript.

### Conflict of interest statement

The authors declare that the research was conducted in the absence of any commercial or financial relationships that could be construed as a potential conflict of interest.
